# Psychological fertility motivations and short-term fertility plans among partnered Chinese adults: differences across parity groups

**DOI:** 10.3389/fpsyg.2026.1917528

**Published:** 2026-10-01

**Authors:** Sijia Wang, Danning Feng

**Affiliations:** School of Ethnology and Sociology, Minzu University of China, Beijing, China

**Keywords:** Emotional Fulfillment Motivation, Family Responsibility Motivation, fertility motivations, parity, short-term fertility plans, Traditional Utility Motivation

## Abstract

**Introduction:**

Fertility motivations reflect the psychological value individuals attach to childbearing and children and provide an important perspective for understanding fertility decision-making. Drawing on Miller’s Traits–Desires–Intentions–Behavior (TDIB) model, this study used data from the China Family Panel Studies (CFPS) to examine the associations between different types of fertility motivations and short-term fertility plans and whether these associations varied by parity.

**Methods:**

The analytical sample comprised 10,671 married or cohabiting adults aged 22–49. Exploratory and confirmatory factor analyses were first conducted to identify and validate the latent structure of fertility motivations. Binary logistic regression models accounting for sampling strata and primary sampling units (PSUs), interaction tests, and average marginal effects were then used for estimation.

**Results:**

Three dimensions of fertility motivation were identified: Emotional Fulfillment Motivation, Traditional Utility Motivation, and Family Responsibility Motivation. In the overall sample, only Traditional Utility Motivation was significantly and positively associated with short-term fertility plans, whereas the overall associations of Emotional Fulfillment Motivation and Family Responsibility Motivation were not statistically significant. Further analyses showed significant parity differences in the associations of both Emotional Fulfillment Motivation and Traditional Utility Motivation with short-term fertility plans, while no significant parity differences were found for Family Responsibility Motivation. Specifically, Emotional Fulfillment Motivation was positively associated with short-term fertility plans only among childless adults. Traditional Utility Motivation was negatively associated with short-term fertility plans among childless adults but positively associated among those with one child and those with two or more children, with the differences primarily occurring between childless adults and parents. Robustness analyses further showed that the parity differences in Traditional Utility Motivation were stable across survey weighting, household-level clustering, and the treatment of multiple respondents within the same household, whereas the parity differences in Emotional Fulfillment Motivation did not reach statistical significance in the sample with valid survey weights.

**Discussion:**

Overall, the associations between fertility motivations and short-term fertility plans vary meaningfully by parity, highlighting the importance of understanding fertility psychology across different stages of family formation.

## 1 Introduction

Since the second half of the twentieth century, declining fertility rates have become a widespread demographic phenomenon across the world. In China, the total fertility rate (TFR) fell below the replacement level of 2.1 during the 1990s (Mu, 2019). According to World Population Prospects 2024, China’s TFR is projected to be only 1.03 in 2026 (United Nations, Department of Economic and Social Affairs, Population Division, 2024). Persistently low fertility has accelerated the onset of population decline in China (Zhang and Wang, 2026). Since 2022, the country has experienced four consecutive years of negative population growth, raising widespread concerns regarding a shrinking labor force and insufficient endogenous drivers of economic development (Tong, 2026).

Research on fertility behavior has traditionally focused on the influence of macro-level structural factors, such as economic development, individualism and gender egalitarian values, employment instability, and income uncertainty (Cai, 2024; Goldin, 2006; McDonald, 2000; Karabchuk, 2020). From a micro-level perspective, childbearing represents one of the most consequential decisions across the life course. It not only shapes family formation and continuity but also influences individuals’ identity construction, sense of meaning in life, and subjective well-being. Fertility decision-making is therefore, at its core, a complex psychological process.

Within psychology, motivation is widely regarded as a central mechanism that initiates, sustains, and directs human behavior. It reflects the underlying sources of behavioral energy, direction, and persistence (Ryan and Deci, 2000). Previous research has suggested that human behavior is not merely a passive response to external circumstances but rather an active process guided by individual needs, goals, and value orientations (Deci and Ryan, 2000; Eccles and Wigfield, 2002). Fertility behavior is no exception. Individuals choose to become parents not only because of social norms or economic constraints, but also because having children can fulfil specific psychological needs and life goals (Hoffman and Hoffman, 1973; Nauck, 2001). In this sense, childbearing can be understood as an intentional behavioral choice that is strongly shaped by underlying fertility motivations. Examining the associations between fertility motivations and short-term fertility plans therefore provides an important avenue for understanding fertility decision-making.

China provides a distinctive cultural context for examining the associations between different fertility motivations and short-term fertility plans. On the one hand, with the expansion of education, rising female labor force participation, and the growing prominence of individualistic values, childbearing has increasingly shifted from a traditional family obligation to a voluntary choice associated with emotional fulfillment and personal self-realization (Feng, 2018; Yang and Wu, 2021). On the other hand, traditional beliefs rooted in Confucian culture and familism—such as expectations of old-age support and family lineage continuation—continue to exert considerable influence on fertility attitudes and behaviors (Sheng and Li, 2023). The coexistence of modern individualistic values and traditional familistic norms makes China a particularly informative setting for examining the associations between different fertility motivations and short-term fertility plans. More importantly, these associations may differ across parity groups, as the same fertility motivation may carry different psychological meanings at different stages of the childbearing process. Although previous research has established the importance of fertility motivations and the value of children, less is known about how different types of fertility motivation are associated with short-term fertility plans and whether these associations differ by parity.

Against this background, this study uses data from the China Family Panel Studies (CFPS) to address three objectives. First, Exploratory Factor Analysis (EFA) and confirmatory factor analysis (CFA) are used to identify and validate the multidimensional structure of fertility motivations. Second, the study examines the associations between different types of fertility motivation and short-term fertility plans. Third, parity is introduced as a moderator to examine whether these associations differ according to respondents’ existing number of children. This study makes two main contributions. First, situated within the Chinese cultural context, it identifies and validates the multidimensional structure of fertility motivations and examines the differential associations between different fertility motivations and short-term fertility plans, using a measure of fertility plans with a clearly defined time horizon. Second, it incorporates parity into the analytical framework linking fertility motivations to short-term fertility plans and examines whether these associations vary across different stages of individuals’ childbearing experience, thereby highlighting life-course heterogeneity in the associations between fertility motivations and short-term fertility plans.

## 2 Theoretical framework and literature review

### 2.1 Theoretical framework: the TDIB model

The Traits–Desires–Intentions–Behavior (TDIB) sequential decision-making model proposed by Miller and colleagues provides an important theoretical framework for understanding the relationship between fertility motivations and subsequent fertility decision-making and behavior. From a psychological perspective, the model conceptualizes fertility decision-making as a dynamic psychological process. Fertility motivations are shaped by biological predispositions, including genetic factors, as well as early life experiences. These motivations provide the psychological basis for the formation of fertility desires and intentions, which are subsequently linked to fertility behavior (Miller, 1994). Within this process, fertility motivations serve as an important psychological mechanism linking individual characteristics to subsequent fertility decision-making and behavior (Miller, 1992; Miller and Pasta, 1995a).

Compared with general fertility desires, fertility plans with a clearly defined time horizon are more closely aligned with concrete behavioral decisions. The Theory of Planned Behavior emphasizes that behavioral intentions are an important antecedent of actual behavior (Ajzen, 1991). Related research has also shown that fertility plans with a clearly defined time horizon are closely associated with subsequent fertility behavior, reflecting the likelihood that individuals will translate their fertility desires into action (Dommermuth et al., 2011). Therefore, examining the associations between fertility motivations and short-term fertility plans can help clarify the relationship between the psychological basis of childbearing and near-term fertility decision-making.

Previous research has found that positive fertility motivations are associated with stronger fertility desires, stronger fertility plans, and higher levels of actual fertility behavior (Miller, 2011; Billari et al., 2009). However, from a life-course perspective, the associations between different fertility motivations and short-term fertility plans may vary depending on individuals’ parity. Existing research has paid relatively little attention to whether these associations differ across parity groups.

### 2.2 Literature review and research hypotheses

#### 2.2.1 Classification of fertility motivations

Fertility motivation refers to individuals’ answers to questions such as “Why do I want to have children?” and “What purposes do I expect childbearing to fulfil in my life?” (Tartakovsky and Mizrahi, 2025). Existing research on fertility motivations has broadly developed along two lines of inquiry: economic and social psychological approaches. Early economic studies focused primarily on the costs and benefits of children, emphasizing their consumption, income-generating, risk-protection, and family-maintenance values and utilities (Becker and Tomes, 1976; Leibenstein, 1975). Social psychologists, by contrast, have approached the issue from the perspective of the actual or potential psychological needs that children may fulfil for parents, giving rise to research traditions such as the value of children (VOC) approach and studies of reasons for childbearing.

As research in this area has developed, fertility motivations, reasons for childbearing, and the value of children have been conceptualized and measured in various ways, resulting in a diverse range of classifications. Despite differences in terminology and theoretical traditions, existing studies broadly point to four major categories of reasons and values associated with childbearing. First, emotional and psychological fulfillment motivations emphasize the love and companionship, sense of fulfillment, personal growth, and enjoyment of raising children associated with childbearing and parent–child relationships. Second, economic and instrumental motivations emphasize the practical functions of children, including financial support, old-age care, and family security. Third, social normative and identity motivations encompass acquiring parental or adult status, fulfilling religious and moral obligations, and meeting social expectations. Fourth, family continuity and relational motivations include continuing the family lineage and transmitting family values and traditions, as well as strengthening couple bonds, enhancing the importance of family, and maintaining ties with relatives (Hoffman and Manis, 1979; Miller, 1995; Liefbroer, 2005; Mayer et al., 2005; Matias and Fontaine, 2013; Guedes et al., 2015).

East Asian societies have been deeply influenced by cultural norms surrounding filial piety, family continuity, and lineage continuation. Some studies have similarly classified the value of children into economic/instrumental, social/normative, and emotional/psychological dimensions (Xia et al., 2024). Other research, however, has suggested that economic values, social norms, and family continuity are closely intertwined and difficult to distinguish, and has instead grouped the value of children into traditional values—including lineage continuation, old-age support, economic contributions to the family, and social prestige—and emotional values emphasizing psychological fulfillment and emotional bonds (Yi and Chen, 2014). Some studies in China have further incorporated culturally specific reasons for childbearing, such as “giving a child a sibling,” “having both a son and a daughter,” and “meeting grandparents’ expectations,” further indicating that the specific forms of fertility motivations are shaped by their sociocultural context (Liu et al., 2025; Hu and Zhao, 2026).

Overall, existing research suggests that fertility motivations comprise multiple dimensions with distinct psychological meanings that may also overlap with one another. It is therefore necessary to identify and examine the dimensional structure of fertility motivations using empirical data. Accordingly, this study proposes the following hypothesis:

H1: Fertility motivations are not unidimensional but have a multidimensional structure.

#### 2.2.2 Fertility motivations and fertility behavior

With socioeconomic development and transformations in family functions, the values attributed to children have also changed substantially. Instrumental values traditionally associated with old-age security, family labor, and economic support have gradually declined, whereas psychological values such as parent–child companionship, emotional fulfillment, and personal growth have become increasingly important (Xia et al., 2024; Kagitcibasi and Ataca, 2005).

However, the extent to which different fertility motivations are socially valued does not necessarily correspond to the strength of their associations with actual fertility decisions. Previous studies have found that although emotional fulfillment, parent–child companionship, and the joy of childrearing are often highly valued reasons for childbearing, the associations of these psychological motivations with ideal family size and actual fertility are inconsistent, with some studies even reporting negative associations (Hoffman and Manis, 1979; Kagitcibasi and Ataca, 2005). By contrast, traditional instrumental and social motivations, such as old-age security, economic support for the family, lineage continuation, and family continuity, remain associated with larger ideal family sizes, stronger fertility intentions, or higher levels of actual fertility (Kim et al., 2005; Chen and Hu, 2020; Song and Hu, 2022), with such associations being particularly evident among rural and migrant populations (Zheng et al., 2005). Related research has also suggested that relational benefits, such as the expansion and strengthening of social relationships through having children, have only limited associations with fertility behavior (Kim et al., 2005).

Based on the above discussion, this study proposes the following hypothesis:

H2: Different types of fertility motivation are differentially associated with individuals’ short-term fertility plans.

#### 2.2.3 A life-course perspective: the moderating role of parity

Life Course Theory posits that individual development is a dynamic process in which different stages of the life course are characterized by distinct developmental tasks, opportunities, and need structures (Elder, 1998; Heckhausen et al., 2010). From this perspective, the associations between fertility motivations and fertility behavior may not remain constant across the life course. Instead, these associations may vary across different stages of individuals’ reproductive lives.

Miller and Pasta (1995b) noted that fertility decision-making is inherently sequential, with each childbearing experience potentially altering individuals’ existing fertility motivations and subsequent fertility desires. Related research has also shown that the associations between different types of the value of children and fertility behavior vary substantially by parity. Drawing on data from multiple countries, Nauck examined the associations between the utility value of children and fertility. His findings indicate that short-term utilities associated with stimulation, enjoyment, and emotional attachment tend to be associated with lower levels of higher-order childbearing, as many of the emotional benefits sought by parents are already achieved following the birth of a first child. Similarly, long-term emotional utilities related to life meaning and psychological fulfillment are associated with lower fertility levels. In contrast, parents who place greater value on the medium-term productive contributions of children and the long-term security benefits they provide are significantly more likely to have larger families (Nauck, 2007, 2014).

Once a psychological need is gratified, its motivational potency to sustain behavior progressively weakens (Maslow, 1943). For childless individuals, childbearing may fulfil important psychological needs related to intimacy, parental identity, and the anticipated enjoyment of parenthood. Consequently, such motivations may effectively promote fertility plans. However, following the birth of a first child, these needs may have already been partially satisfied, reducing the marginal motivational value of emotional rewards associated with additional childbearing. By contrast, motivations emphasising family continuity, old-age security, and intergenerational support possess more enduring and cumulative characteristics. As families move through successive stages of the life course, the perceived benefits of having multiple children—such as risk diversification, family support, and intergenerational reciprocity—may become increasingly salient. Consequently, these motivations may exert a stronger influence on fertility plans at higher parity levels (Nauck, 2014).

Based on the above discussion, the following hypotheses are proposed:

H3: The associations between different types of fertility motivation and short-term fertility plans differ across parity groups.

## 3 Materials and methods

### 3.1 Data and sample

This study used data from the China Family Panel Studies (CFPS), a nationally representative longitudinal survey conducted by the Institute of Social Science Survey (ISSS) at Peking University. The CFPS employs a multistage stratified probability sampling design and covers 25 provinces, municipalities, and autonomous regions across mainland China. The survey collects extensive information on demographic characteristics, family relationships, socioeconomic conditions, and individual attitudes, providing a valuable resource for research on population, family, and social change in contemporary China.

This study uses data from the 2020 and 2022 waves. It is important to note that the fertility motivation module was not administered to exactly the same target population in the two waves. In 2020, the module was administered primarily to respondents who met the relevant age criterion. In 2022, it was administered primarily to age-eligible respondents who had not completed the fertility motivation module in 2020. Thus, the 2022 fertility motivation data primarily provide supplementary observations for respondents who had not completed the corresponding module in 2020, rather than constituting an independently drawn 2022 cross-sectional sample or a follow-up assessment of the same respondents surveyed in 2020. Because this study focuses on the contemporaneous associations between respondents’ fertility motivations and their plans to have a child within the next 2 years, the 2020 sample and the supplementary 2022 sample were pooled to construct the analytical sample. Survey wave was included as a covariate in the regression models to account for average differences between the two analytic samples.

Sample selection was conducted separately for the two survey waves. According to the CFPS questionnaire design, the fertility motivation scale was administered only to respondents aged over 21, whereas the fertility planning question was administered only to married or cohabiting respondents under the age of 50. In addition, the 2022 fertility motivation module incorporated routing based on respondents’ completion of the corresponding scale in 2020; in principle, respondents who had completed the scale in 2020 were not surveyed again in 2022. Based on the above eligibility criteria and questionnaire routing rules, unavailable observations for the relevant variables were classified according to their source. Non-administration resulting from survey eligibility or questionnaire routing was treated as structural non-administration, whereas refusals, “do not know” responses, and other invalid responses were treated as item nonresponse. System-missing cases for which the specific reason could not be further determined were identified separately. A complete-case analysis was then applied, retaining only respondents with valid observations on fertility plans, fertility motivations, parity, and all covariates in the final analytical sample. The detailed sample-selection process is presented in Supplementary Table S1.

After sample selection was completed separately for the two waves, the pooled data were further checked for repeated observations across waves using respondents’ unique identifiers (pid). Five respondents appeared in both the 2020 and 2022 samples. Because the pooled analysis was designed to include each respondent as a single independent observation, we retained the 2020 record and excluded the corresponding 2022 record for these respondents to avoid duplicate contributions from the same individual. After applying the eligibility criteria, handling item nonresponse, and removing repeated observations, the final analytical sample consisted of 10,671 respondents, including 8,645 from 2020 and 2,026 from 2022.

### 3.2 Measures

#### 3.2.1 Fertility motivation

Fertility motivation was measured using a nine-item scale assessing respondents’ reasons for having children, which was included in the adult questionnaire of the 2020 and 2022 waves of the China Family Panel Studies (CFPS). Respondents were asked to indicate the extent to which they agreed with the following reasons for childbearing: (1) to have help in old age; (2) to continue the family line; (3) to help the family financially; (4) for the joy of watching children grow up; (5) for the happiness of having children around; (6) for the joy of having a baby; (7) to make the family more important; (8) to enhance a sense of responsibility; (9) to increase kinship ties. All items were measured on a five-point Likert scale. To ensure consistency in scale direction, the original coding was recoded such that higher scores indicated stronger endorsement of each motivation (1 = strongly disagree, 2 = disagree, 3 = neither agree nor disagree, 4 = agree, and 5 = strongly agree).

To identify the underlying dimensional structure of the nine items, we further conducted factor analysis.

#### 3.2.2 Short-term fertility plans

Following research applying the Theory of Planned Behavior to fertility decision-making (Ajzen and Klobas, 2013), this study measures short-term fertility plans using the CFPS question, “Do you plan to have a child within the next two years?” The question specifies a clear time horizon and captures respondents’ near-term fertility plans. Responses were coded as 1 for “yes” and 0 for “no.”

#### 3.2.3 Parity

Parity was measured based on the number of children respondents had at the time of the survey. The original measure was a count variable. Given that the transition from childlessness to first birth and the transition from one child to subsequent childbearing represent distinct stages of the family life course and different fertility decision-making contexts, parity was recoded into three categories: childless, one child, and two or more children.

#### 3.2.4 Covariates

Drawing on previous research and the availability of relevant variables in the CFPS, a range of individual- and family-level covariates were included in the analyses. These variables comprised age, age squared, gender, household registration status (hukou), self-rated health, educational attainment, employment status, household per capita income, and region of residence. Age was measured in years at the time of the survey, and an age-squared term was included to account for potential non-linear age effects. Household economic conditions were measured using the natural logarithm of annual household per capita income. The remaining covariates were coded into categorical variables according to analytical requirements.

Table 1 presents the unweighted and survey-weighted descriptive statistics for the analytical sample. The unweighted statistics are based on the full analytical sample, whereas the weighted statistics were calculated using the CFPS individual cross-sectional weights for the corresponding survey wave. In the unweighted sample, 12.86% of respondents reported plans to have a child within the next 2 years. This proportion differed substantially across parity groups, at 52.20% among childless respondents, 14.36% among respondents with one child, and 4.98% among those with two or more children. The survey-weighted estimates showed slightly lower proportions overall and across parity groups. Overall, although the weighted and unweighted estimates differed slightly in the distributions of some variables, the main distributional characteristics of the sample were broadly similar.

**Table 1:** Weighted and unweighted descriptive statistics of the analytical sample.

Variable	Full analytical sample: unweighted (*N* = 10,671)	Sample with valid survey weights: weighted (*N* = 8,326)
Fertility motivation items, Mean (SD)		
To have help in old age	3.58 (1.04)	3.56 (1.03)
To continue the family line	3.37 (1.09)	3.35 (1.08)
To help the family financially	2.81 (1.09)	2.82 (1.08)
For the joy of watching children grow up	4.07 (0.66)	4.05 (0.64)
For the happiness of having children around	4.09 (0.63)	4.05 (0.63)
For the joy of having a baby	4.05 (0.65)	4.03 (0.64)
To make the family more important	3.90 (0.81)	3.88 (0.79)
To enhance a sense of responsibility	3.95 (0.72)	3.93 (0.70)
To increase kinship ties	3.72 (0.88)	3.72 (0.86)
Age, Mean (SD)	36.99 (7.14)	38.86 (6.95)
Log household per capita income, Mean (SD)	9.95 (1.00)	9.96 (1.10)
Parity, %		
Childless	9.39	9.39
One child	36.74	36.90
Two or more children	53.87	53.71
Gender, %		
Female	53.38	50.49
Male	46.62	49.51
Educational attainment, %		
Primary school or below	20.99	22.41
Junior high school	36.38	32.98
Senior high/Vocational	18.14	19.75
Junior college or above	24.49	24.85
Hukou status, %		
Non-agricultural hukou	28.14	31.42
Agricultural hukou	71.86	68.58
Health status, %		
Unhealthy	14.86	16.16
Healthy	85.14	83.84
Employment status, %		
Not employed	13.60	11.62
Employed	86.40	88.38
Region, %		
East	33.50	35.80
Central	25.57	30.55
West	30.33	24.64
Northeast	10.60	9.02
Survey wave, %		
2020	81.01	83.79
2022	18.99	16.21
Plans to have a child within the next 2 years, %	12.86	9.95
By parity		
Childless	52.20	48.06
One child	14.36	9.58
Two or more children	4.98	3.53
By survey wave		
2020	11.88	9.49
2022	17.03	12.30

Continuous variables are presented as means (standard deviations), and categorical variables as percentages. Unweighted estimates are based on the full analytical sample (*N* = 10,671); weighted estimates are based on respondents with valid CFPS individual cross-sectional survey weights (*N* = 8,326).

### 3.3 Analytical strategy

All statistical analyses were conducted using Stata/MP 18.0. The analyses included assessment of the measurement structure of fertility motivations, binary logistic regression, tests of heterogeneity across parity groups, and robustness analyses.

First, to examine the underlying structure of the nine CFPS fertility motivation items, the final analytical sample was randomly divided into two approximately equal subsamples, while maintaining a broadly similar distribution of the 2020 and 2022 observations across the two subsamples. One subsample was used for Exploratory Factor Analysis (EFA) and the other for Confirmatory Factor Analysis (CFA). In the EFA, latent factors were extracted using the principal-factor method, and the number of factors was determined with the aid of parallel analysis; Promax oblique rotation was then applied. CFA was used to validate the measurement structure, and multi-group CFA was further conducted to assess measurement invariance across parity groups and survey waves. Based on the validated structure, fertility motivation measures were constructed and standardized for use in the subsequent regression analyses.

Second, binary logistic regression models were used to examine the associations between fertility motivations and short-term fertility plans. The main models were estimated using the full analytical sample and accounted for the complex survey design using the CFPS sampling strata and primary sampling units (PSUs). To assess heterogeneity across parity groups, interaction terms between fertility motivations and parity were subsequently introduced, and average marginal effects (AMEs) were used to interpret differences across parity groups.

Finally, to assess the robustness of the findings, additional analyses were conducted using wave-specific cross-sectional survey weights, household-level clustering, and a sample retaining only one respondent per household-wave. Wave-stratified models and fertility motivation × survey wave interaction terms were also estimated to assess the consistency of the findings across survey waves.

## 4 Results

### 4.1 Measurement results for fertility motivations

#### 4.1.1 Exploratory Factor Analysis

A total of 5,335 respondents were included in the Exploratory Factor Analysis (EFA). Tests of factorability yielded a Kaiser–Meyer–Olkin (KMO) value of 0.814, and Bartlett’s test of sphericity was statistically significant [*χ*^2^(36) = 13,512.13, *p* < 0.001], indicating that the correlation structure among the items was suitable for factor analysis. Parallel analysis supported the retention of three common factors. Factors were extracted using the principal-factor method, followed by Promax oblique rotation. The complete factor loading matrix for the nine items across the three factors is presented in Table 2.

**Table 2:** Results of the exploratory factor analysis of fertility motivations.

Item	Emotional Fulfillment Motivation	Family Responsibility Motivation	Traditional Utility Motivation	Communality (*h*^2^)
1. To have help in old age	0.027	−0.025	**0.644**	0.407
2. To continue the family line	−0.017	0.022	**0.674**	0.464
3. To help the family financially	−0.007	0.042	**0.615**	0.405
4. For the joy of watching children grow up	**0.715**	−0.052	0.025	0.477
5. For the happiness of having children around	**0.729**	0.025	0.005	0.556
6. For the joy of having a baby	**0.680**	0.102	−0.027	0.545
7. To make the family more important	0.112	**0.545**	0.030	0.403
8. To enhance a sense of responsibility	0.097	**0.602**	−0.023	0.428
9. To increase kinship ties	0.004	**0.582**	0.081	0.397
Cronbach’s α	0.797	0.702	0.730	
KMO = 0.814. Bartlett’s *χ*^2^(36) = 13512.13, *p* < 0.001				

Bold values indicate the primary factor loading of each item.

The EFA results revealed three dimensions with clearly distinguishable substantive content. Items 4–6 loaded primarily on Factor 1, reflecting the psychological fulfillment and emotional companionship associated with having and raising children; this factor was therefore labeled Emotional Fulfillment Motivation. Items 7–9 loaded primarily on Factor 2 and captured the importance of family, a sense of responsibility, and kinship ties; this factor was labeled Family Responsibility Motivation. Items 1–3 loaded primarily on Factor 3, reflecting the traditional functions of children in Chinese culture, including old-age support, family lineage continuation, and financial support; this factor was therefore labeled Traditional Utility Motivation. Cronbach’s α values for the three dimensions were 0.797, 0.702, and 0.730, respectively, indicating acceptable internal consistency.

#### 4.1.2 Confirmatory factor analysis

To further evaluate the three-factor structure identified by the EFA, Confirmatory Factor Analysis (CFA) was conducted using the other independent subsample. Based on the EFA results, the model specified three latent variables—Traditional Utility Motivation, Emotional Fulfillment Motivation, and Family Responsibility Motivation—measured by Items 1–3, 4–6, and 7–9, respectively. The standardized factor loadings of all items on their corresponding latent variables ranged from 0.660 to 0.787 and were statistically significant (all *p* < 0.001; see Table 3). The three-factor model demonstrated a good fit to the data [*χ*^2^(24) = 271.134, *p* < 0.001; CFI = 0.983; TLI = 0.974; RMSEA = 0.044, 90% CI (0.039, 0.049); SRMR = 0.026], providing support for the measurement structure in the independent subsample.

**Table 3:** Results of the three-factor confirmatory factor analysis of the nine items.

Item	Traditional Utility Motivation	Emotional Fulfillment Motivation	Family Responsibility Motivation
1. To have help in old age	0.670	—	—
2. To continue the family line	0.728	—	—
3. To help the family financially	0.676	—	—
4. For the joy of watching children grow up	—	0.721	—
5. For the happiness of having children around	—	0.787	—
6. For the joy of having a baby	—	0.781	—
7. To make the family more important	—	—	0.683
8. To enhance a sense of responsibility	—	—	0.705
9. To increase kinship ties	—	—	0.660
CFA model fit	*χ*^2^(24) = 271.134, *p* < 0.001; CFI = 0.983; TLI = 0.974; RMSEA = 0.044, 90% CI [0.039, 0.049]; SRMR = 0.026		

Standardized factor loadings are reported. All factor loadings were statistically significant at *p* < 0.001.

Considering that the subsequent analyses focused on whether the associations between fertility motivations and short-term fertility plans differed by parity, and that the data were drawn from the 2020 and 2022 survey waves, we further tested the measurement invariance of the three-factor structure across parity groups and survey waves. Configural invariance and metric invariance models were estimated separately to assess whether the three-factor structure and item factor loadings were comparable across parity groups and survey waves.

For the invariance tests across parity groups, the configural invariance model showed a good fit to the data (CFI = 0.982, TLI = 0.973, RMSEA = 0.045, SRMR = 0.033). The metric invariance model also demonstrated a good fit (CFI = 0.980, TLI = 0.974, RMSEA = 0.044, SRMR = 0.040), with only small changes in model fit (ΔCFI = −0.002, ΔRMSEA = −0.001, ΔSRMR = 0.007), supporting metric invariance across parity groups. Similar results were obtained for the invariance tests across survey waves. Both the configural invariance model (CFI = 0.981, TLI = 0.971, RMSEA = 0.047, SRMR = 0.028) and the metric invariance model (CFI = 0.981, TLI = 0.974, RMSEA = 0.044, SRMR = 0.030) showed good fit, and changes in model fit were likewise small (ΔCFI = 0.000, ΔRMSEA = −0.003, ΔSRMR = 0.002), supporting metric invariance across survey waves.

Overall, the CFA conducted in the independent subsample and the measurement invariance tests across parity groups and survey waves provided consistent support for the three-factor measurement structure of the nine fertility motivation items. These results provide a measurement basis for using the three dimensions of fertility motivation in the subsequent analyses of their associations with short-term fertility plans and differences across parity groups (see Table 4).

**Table 4:** Measurement invariance tests across parity groups and survey waves.

Grouping variable	Model	*χ*^2^(df)	CFI	TLI	RMSEA	90% CI	SRMR	ΔCFI	ΔRMSEA	ΔSRMR
Parity	Configural	330.494 (72)	0.982	0.973	0.045	[0.040, 0.050]	0.033	—	—	—
	Metric	371.491 (84)	0.980	0.974	0.044	[0.039, 0.048]	0.040	−0.002	−0.001	+0.007
Survey wave	Configural	325.087 (48)	0.981	0.971	0.047	[0.042, 0.051]	0.028	—	—	—
	Metric	329.883 (54)	0.981	0.974	0.044	[0.039, 0.048]	0.030	0.000	−0.003	+0.002

Configural, configural invariance model; Metric, metric invariance model. Δ indices represent changes in fit indices from the corresponding configural model to the metric model.

Taken together, the results of the EFA, CFA, and measurement invariance tests supported a three-dimensional structure of fertility motivations comprising Emotional Fulfillment Motivation, Family Responsibility Motivation, and Traditional Utility Motivation. The three-factor structure also demonstrated good stability across parity groups and survey waves, providing support for H1. Based on this validated structure, unit-weighted composite scoring was used to calculate the mean score of the items corresponding to each dimension. The resulting scores were then standardized to have a mean of 0 and a standard deviation of 1, yielding three fertility motivation measures for use in the subsequent regression analyses.

### 4.2 Overall associations between fertility motivations and short-term fertility plans

Table 5 presents the results of the binary logistic regression analysis examining the associations between fertility motivations and short-term fertility plans. The model simultaneously included Emotional Fulfillment Motivation, Traditional Utility Motivation, and Family Responsibility Motivation, while controlling for parity, survey wave, and other sociodemographic and socioeconomic characteristics. The results showed that, in the full sample, Traditional Utility Motivation was significantly and positively associated with short-term fertility plans (*b* = 0.100, SE = 0.034, *p* < 0.01). Holding other variables constant, respondents with higher scores on Traditional Utility Motivation were more likely to report plans to have a child within the next 2 years. In contrast, the overall associations of Emotional Fulfillment Motivation and Family Responsibility Motivation with short-term fertility plans were not statistically significant.

**Table 5:** Binary logistic regression results for fertility motivations and short-term fertility plans.

Variable	Model 1
Emotional Fulfillment Motivation	0.002 (0.034)
Traditional Utility Motivation	0.100** (0.034)
Family Responsibility Motivation	0.009 (0.039)
*Control variables*	
Parity (Ref: Childless)	
One child	−1.861*** (0.112)
Two or more children	−3.058*** (0.150)
2022 (Ref: 2020)	0.428*** (0.096)
Male (Ref: Female)	0.418*** (0.067)
Age	0.365*** (0.089)
Age squared	−0.008*** (0.001)
Educational attainment (Ref: Primary school or below)	
Junior high	−0.391* (0.152)
Senior high/Vocational	−0.430** (0.157)
Junior college or above	−0.088 (0.176)
Agricultural hukou (Ref: Non-agricultural hukou)	0.255** (0.087)
Healthy (Ref: Unhealthy)	0.032 (0.126)
Log household per capita income	0.084 (0.049)
Employed (Ref: Not employed)	0.080 (0.111)
Region (Ref: East)	
Central	−0.105 (0.110)
West	0.060 (0.096)
Northeast	−0.453* (0.196)
Intercept	−4.240** (1.506)
*N*	10,671
Strata	6
PSUs	176
Design df	170
Model F	59.68***

Coefficients are reported with standard errors in parentheses. The main model accounts for the CFPS sampling strata and primary sampling units (PSUs). ***p < 0.001, **p < 0.01, *p < 0.05.

Parity was significantly associated with short-term fertility plans. Compared with childless respondents, respondents with one child (*b* = −1.861, SE = 0.112, *p* < 0.001) and those with two or more children (*b* = −3.058, SE = 0.150, *p* < 0.001) were significantly less likely to report plans to have a child within the next 2 years. In addition, gender, age, age squared, and some categories of educational attainment, hukou status, and region were significantly associated with short-term fertility plans.

To further assess the magnitude of these associations, we calculated the average marginal effects (AMEs) of the three fertility motivation dimensions. The results showed that a one-standard-deviation increase in Traditional Utility Motivation was associated with an average increase of approximately 0.79 percentage points in the predicted probability of reporting plans to have a child within the next 2 years [AME = 0.0079, *p* < 0.01, 95% CI (0.0026, 0.0132)]. In contrast, the AMEs of Emotional Fulfillment Motivation and Family Responsibility Motivation were not statistically significant. Overall, the three fertility motivation dimensions exhibited different patterns of association with short-term fertility plans, with only Traditional Utility Motivation showing a significant overall positive association. These findings provide support for H2.

### 4.3 Moderating role of parity in the associations between fertility motivations and short-term fertility plans

To examine whether the associations between different fertility motivations and short-term fertility plans varied by parity, we first included the interactions between all three fertility motivation dimensions and parity simultaneously in the full model and conducted separate joint Wald tests. The results showed that the interactions of Emotional Fulfillment Motivation [*F*(2,169) = 3.21, *p* = 0.043] and Traditional Utility Motivation [*F*(2,169) = 12.99, *p* < 0.001] with parity were statistically significant. In contrast, the interaction between Family Responsibility Motivation and parity was not statistically significant [*F*(2,169) = 0.58, *p* = 0.560]. Accordingly, the subsequent model retained the main effect of Family Responsibility Motivation and focused on the interactions of Emotional Fulfillment Motivation and Traditional Utility Motivation with parity. Average marginal effects were further used to examine differences across parity groups.

Specifically, with the childless group as the reference category, the interaction terms between Emotional Fulfillment Motivation and the one-child and two-or-more-child groups were both significantly negative [*b* = −0.192, *p* = 0.034, 95% CI (−0.368, −0.015); *b* = −0.219, *p* = 0.024, 95% CI (−0.409, −0.030), respectively], indicating significant parity differences in the association between Emotional Fulfillment Motivation and short-term fertility plans. Further examination of group-specific associations showed that Emotional Fulfillment Motivation was significantly and positively associated with short-term fertility plans among childless adults [*b* = 0.162, SE = 0.078, *p* = 0.038, 95% CI (0.009, 0.316)], whereas the associations were not statistically significant among adults with one child or those with two or more children (both *p* > 0.05). Pairwise comparisons of the average marginal effects further showed significant differences between the childless group and both the one-child group and the two-or-more-child group (ΔAME = −0.0305, *p* = 0.028; ΔAME = −0.0301, *p* = 0.019, respectively), whereas the difference between the one-child and two-or-more-child groups was not statistically significant (*p* = 0.941). Overall, the parity differences in the association of Emotional Fulfillment Motivation with short-term fertility plans were primarily concentrated between childless adults and parents, with no significant difference found between adults with one child and those with two or more children.

Traditional Utility Motivation exhibited a different pattern across parity groups. With the childless group as the reference category, its interaction terms with the one-child and two-or-more-child groups were both significantly positive [*b* = 0.453, *p* < 0.001, 95% CI (0.273, 0.632); *b* = 0.450, *p* < 0.001, 95% CI (0.235, 0.664), respectively], indicating significant parity differences in the association between Traditional Utility Motivation and short-term fertility plans. Further examination of group-specific associations showed that Traditional Utility Motivation was significantly and negatively associated with short-term fertility plans among childless adults [*b* = −0.258, SE = 0.083, *p* = 0.002, 95% CI (−0.421, −0.094)], whereas it was significantly and positively associated with short-term fertility plans among adults with one child and those with two or more children (both *p* < 0.01). Pairwise comparisons of the average marginal effects further showed significant differences between the childless group and both the one-child and two-or-more-child groups (ΔAME = 0.0647 and 0.0525, respectively; both *p* < 0.001), whereas the difference between the one-child and two-or-more-child groups was not statistically significant (*p* = 0.066). Overall, the parity differences in the association of Traditional Utility Motivation with short-term fertility plans were likewise primarily concentrated between childless adults and parents. Unlike Emotional Fulfillment Motivation, however, the direction of the association reversed between childless adults and parents. These findings provide partial support for H3 (see Table 6).

**Table 6:** Logistic regression results for fertility motivations, parity, and their interactions with short-term fertility plans.

Variable	Model 2	95% CI
Emotional Fulfillment Motivation	0.162* (0.078)	[0.009, 0.316]
One child × Emotional Fulfillment Motivation	−0.192* (0.090)	[−0.368, −0.015]
Two or more children × Emotional Fulfillment Motivation	−0.219* (0.096)	[−0.409, −0.030]
Traditional Utility Motivation	−0.258** (0.083)	[−0.421, −0.094]
One child × Traditional Utility Motivation	0.453*** (0.091)	[0.273, 0.632]
Two or more children × Traditional Utility Motivation	0.450*** (0.109)	[0.235, 0.664]
Family Responsibility Motivation	0.010 (0.040)	[−0.068, 0.088]
*Control Variables*		
Parity (Ref: Childless)		
One child	−1.768*** (0.111)	[−1.988, −1.548]
Two or more children	−3.006*** (0.149)	[−3.300, −2.712]
2022 (Ref: 2020)	0.442*** (0.097)	[0.250, 0.634]
Male (Ref: Female)	0.419*** (0.067)	[0.286, 0.552]
Age	0.357*** (0.089)	[0.181, 0.532]
Age squared	−0.008*** (0.001)	[−0.010, −0.005]
Educational attainment (Ref: Primary school or below)		
Junior high	−0.382* (0.148)	[−0.674, −0.089]
Senior high/Vocational	−0.420** (0.153)	[−0.722, −0.117]
Junior college or above	−0.080 (0.171)	[−0.418, 0.258]
Agricultural hukou (Ref: Non-agricultural hukou)	0.265** (0.088)	[0.092, 0.439]
Healthy (Ref: Unhealthy)	0.020 (0.126)	[−0.230, 0.269]
Log household per capita income	0.076 (0.050)	[−0.022, 0.174]
Employed (Ref: Not employed)	0.068 (0.112)	[−0.154, 0.289]
Region (Ref: East)		
Central	−0.096 (0.111)	[−0.314, 0.122]
West	0.057 (0.096)	[−0.132, 0.246]
Northeast	−0.477* (0.196)	[−0.864, −0.091]
Intercept	−4.096** (1.510)	[−7.076, −1.117]
*N*	10,671	

Standard errors in parentheses are linearized standard errors adjusted for sampling strata and primary sampling units (PSUs); 95% CI denotes the 95% confidence interval. ***p < 0.001, **p < 0.01, *p < 0.05.

### 4.4 Average marginal effects across parity groups

To further illustrate the magnitude of the associations between fertility motivations and short-term fertility plans across parity groups, we calculated adjusted average predicted probabilities and average marginal effects (AMEs). The adjusted average predicted probabilities of reporting plans to have a child within the next 2 years were 41.67, 14.77, and 5.31% for childless adults, adults with one child, and adults with two or more children, respectively. Based on these estimates, the AMEs of the three fertility motivation dimensions were further calculated for each parity group (see Figure 1).

**Figure 1:**
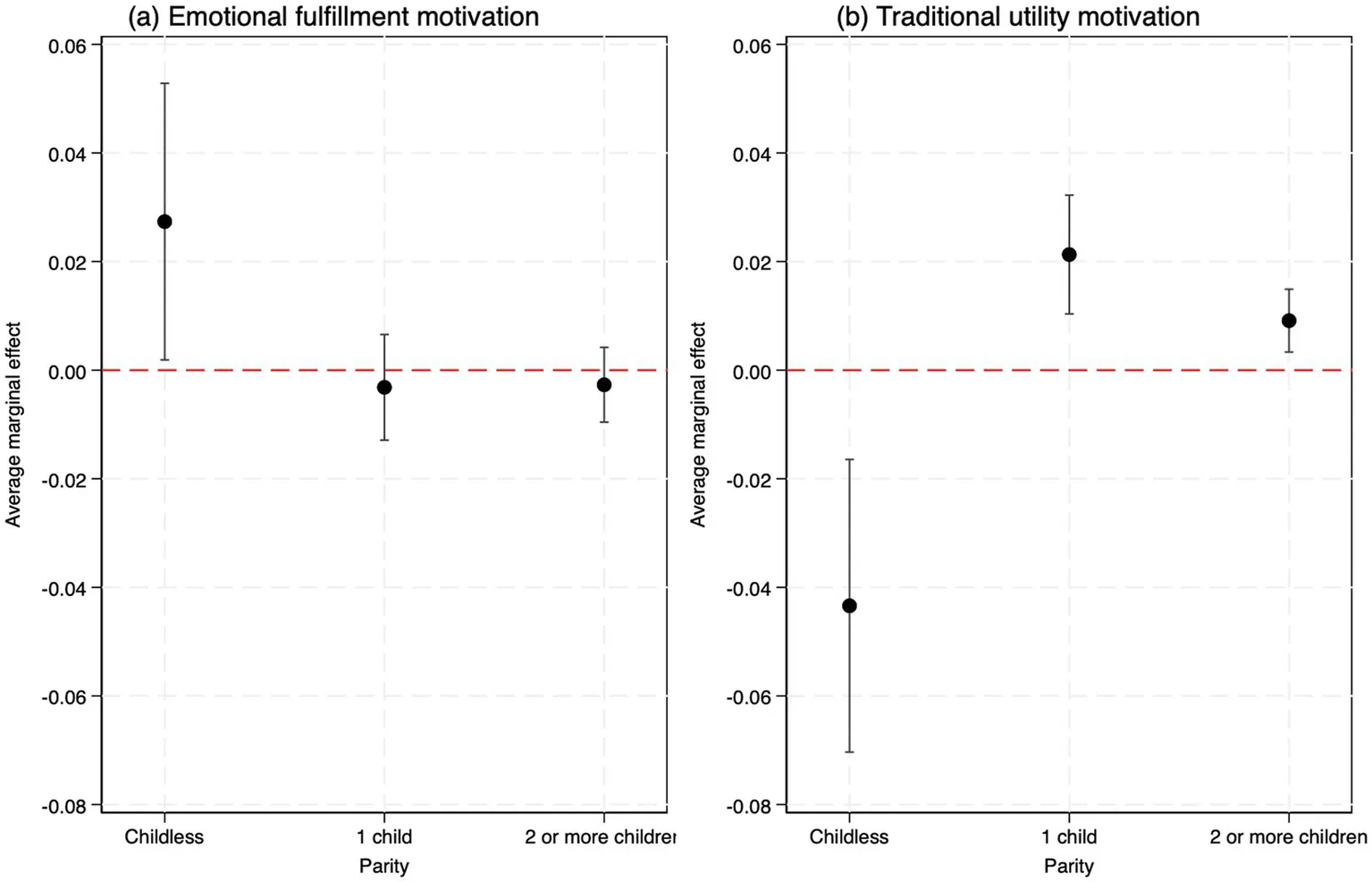
Average marginal effects of fertility motivations across parity groups. Dots represent point estimates of the average marginal effects, vertical lines represent 95% confidence intervals, and the horizontal dashed line indicates an average marginal effect of zero. When the 95% confidence interval includes zero, the average marginal effect is not statistically significant at the 5% level.

Among childless adults, a one-standard-deviation increase in Emotional Fulfillment Motivation was associated with an average increase of approximately 2.74 percentage points in the predicted probability of reporting plans to have a child within the next 2 years [AME = 0.0274, SE = 0.0129, *p* = 0.035, 95% CI (0.0019, 0.0528)]. Among adults with one child and those with two or more children, the corresponding AMEs were −0.32 and −0.27 percentage points, respectively, and neither was statistically significant (*p* = 0.521 and *p* = 0.441, respectively). These results indicate that the positive association between Emotional Fulfillment Motivation and short-term fertility plans was primarily observed among childless adults.

Traditional Utility Motivation exhibited parity differences in the opposite direction. Among childless adults, a one-standard-deviation increase in Traditional Utility Motivation was associated with an average decrease of approximately 4.34 percentage points in the predicted probability of reporting plans to have a child within the next 2 years [AME = −0.0434, SE = 0.0137, *p* = 0.002, 95% CI (−0.0703, −0.0164)]. In contrast, among adults with one child and those with two or more children, the predicted probability increased by approximately 2.13 percentage points [AME = 0.0213, SE = 0.0055, *p* < 0.001, 95% CI (0.0104, 0.0322)] and 0.91 percentage points [AME = 0.0091, SE = 0.0029, *p* = 0.002, 95% CI (0.0033, 0.0149)], respectively. Thus, the association between Traditional Utility Motivation and short-term fertility plans was in opposite directions among childless adults and parents.

### 4.5 Robustness checks

To examine whether the main findings were sensitive to the sampling design and within-household dependence, additional analyses were conducted using survey weighting, unweighted estimation in the same analytical sample, household-level clustering, and a sample retaining only one respondent per household-wave. The survey-weighted model applied wave-specific cross-sectional weights and accounted for sampling strata and primary sampling units (PSUs); an unweighted model was also estimated using the same 8,326 respondents. The directions of the interaction terms were consistent with those in the main analysis in both models. The joint test of the Traditional Utility Motivation × parity interaction remained statistically significant in both the weighted and unweighted models (both *p* < 0.001), whereas the joint test of the Emotional Fulfillment Motivation × parity interaction was not statistically significant in either model (weighted: *p* = 0.088; unweighted: *p* = 0.109). These results indicate that the joint test for the Emotional Fulfillment Motivation × parity interaction was no longer statistically significant when the analytical sample was restricted to respondents with valid survey weights, and applying survey weights to the same sample did not alter this conclusion.

The main findings also remained broadly stable after accounting for potential correlations among respondents from the same household. When cluster-robust standard errors at the household-wave level were used, the joint interactions of both Emotional Fulfillment Motivation and Traditional Utility Motivation with parity were statistically significant (*p* = 0.046 and *p* < 0.001, respectively). After retaining only one respondent per household-wave, both joint tests remained statistically significant (*p* = 0.037 and *p* < 0.001, respectively). These results suggest that the parity differences identified in the main analysis remained evident after accounting for within-household correlations and the inclusion of multiple respondents from the same household.

Overall, the parity-moderating effect of Traditional Utility Motivation was relatively stable across model specifications. Although the parity-moderating effect of Emotional Fulfillment Motivation remained supported in some robustness analyses, it did not reach statistical significance in the sample with valid survey weights. This finding should therefore be interpreted with caution.

Given the differences in questionnaire routing and sample composition between the 2020 and 2022 survey waves, we further examined the cross-wave stability of the pooled results using wave-stratified regressions and interaction terms between fertility motivations and survey wave. The results are presented in Table 7, Panel B. The interactions of Emotional Fulfillment Motivation and Traditional Utility Motivation with survey wave were not statistically significant (*p* = 0.553 and *p* = 0.642, respectively), indicating no significant cross-wave heterogeneity in the associations of these two motivation dimensions with short-term fertility plans. Family Responsibility Motivation, however, exhibited some cross-wave heterogeneity: its association was not statistically significant in the 2020 sample but was significantly positive in the 2022 sample, and the test of the cross-wave difference was statistically significant (*p* = 0.029). It should be noted that this cross-wave heterogeneity does not involve the moderating role of parity examined in this study. As shown in the preceding analyses, the interaction between Family Responsibility Motivation and parity was not statistically significant, whereas the parity differences in the associations of Emotional Fulfillment Motivation and Traditional Utility Motivation constitute the main findings of this study. Thus, the cross-wave difference in Family Responsibility Motivation indicates some heterogeneity across survey waves in its overall association with short-term fertility plans, but does not alter the main conclusion regarding parity differences in the associations of the two focal fertility motivation dimensions.

**Table 7:** Results of robustness checks.

Panel A robustness checks of the core parity moderation results				
Variable	Model 3: survey-weighted	Model 4: weight-eligible sample, unweighted	Model 5: household-wave clustered	Model 6: one respondent per household-wave
Emotional Fulfillment Motivation	0.167 (0.100)	0.172* (0.082)	0.162* (0.072)	0.174* (0.080)
One child × Emotional Fulfillment Motivation	−0.180 (0.133)	−0.150 (0.112)	−0.192* (0.087)	−0.234* (0.104)
Two or more children × Emotional Fulfillment Motivation	−0.255* (0.118)	−0.218* (0.108)	−0.219* (0.100)	−0.224* (0.109)
Traditional Utility Motivation	−0.168 (0.115)	−0.322** (0.098)	−0.258** (0.081)	−0.279** (0.091)
One child × Traditional Utility Motivation	0.273 (0.140)	0.410*** (0.108)	0.453*** (0.094)	0.533*** (0.107)
Two or more children × Traditional Utility Motivation	0.599*** (0.150)	0.518*** (0.136)	0.450*** (0.103)	0.504*** (0.130)
Family Responsibility Motivation	−0.004 (0.069)	0.043 (0.049)	0.010 (0.039)	0.018 (0.047)
Other control variables	Controlled	Controlled	Controlled	Controlled
Joint Wald tests				
Emotional Fulfillment Motivation × Parity	*p* = 0.088	*p* = 0.109	*p* = 0.046	*p* = 0.037
Traditional Utility Motivation × Parity	*p* < 0.001	*p* < 0.001	*p* < 0.001	*p* < 0.001
*N*	8,326	8,326	10,671	7,038

Panel B wave-stratified regressions and cross-wave difference tests				
Variable	2020	2022	Motivation × 2022	Cross-wave difference test *p* value
Emotional Fulfillment Motivation	0.001 (0.042)	0.020 (0.073)	0.049 (0.083)	*p* = 0.553
Traditional Utility Motivation	0.090 (0.046)	0.137 (0.072)	−0.042 (0.091)	*p* = 0.642
Family Responsibility Motivation	−0.032 (0.046)	0.160* (0.065)	0.168* (0.076)	*p* = 0.029
Other control variables	Controlled	Controlled	Controlled	—
*N*	8,645	2,026	10,671	—

In Panel A, the weighted and unweighted estimates are based on the same 8,326 respondents. In Panel B, 2020 is the reference wave, and cross-wave differences are assessed using adjusted Wald tests. ***p < 0.001, **p < 0.01, *p < 0.05.

## 5 Discussion and conclusion

This study draws on Miller’s (1994) Traits–Desires–Intentions–Behavior (TDIB) model and data from the China Family Panel Studies (CFPS) to examine the associations between different types of fertility motivation and individuals’ short-term fertility plans among married or cohabiting Chinese adults aged 22–49, as well as whether these associations differ across parity groups. The TDIB model emphasizes a sequential process of fertility decision-making, in which fertility motivations provide a psychological basis for the formation of fertility desires and intentions, which are subsequently linked to fertility behavior. This framework therefore provides an important theoretical basis for understanding the relationship between individuals’ fertility psychology and fertility decision-making.

Regarding the measurement structure of fertility motivations, this study identified three dimensions—Emotional Fulfillment Motivation, Traditional Utility Motivation, and Family Responsibility Motivation—and further validated this structure through confirmatory factor analysis in an independent subsample. This finding is consistent with previous research in recognizing the multidimensional nature of fertility motivations, while revealing a more differentiated dimensional structure. Previous studies using CFPS data have classified the fertility motivation scale into two dimensions (Zuo and Huang, 2025; Chen and Hu, 2020). Building on this research, the present study pooled the 2020 CFPS sample with the supplementary 2022 sample and identified three correlated but conceptually distinct dimensions: Emotional Fulfillment Motivation, Traditional Utility Motivation, and Family Responsibility Motivation. The findings thus further refine the internal structure of fertility motivations beyond the two-dimensional classification used in previous research, suggesting that different reasons for childbearing may involve more nuanced psychological distinctions. Furthermore, by examining the associations between different fertility motivations and short-term fertility plans and their differences across parity groups, this study provides more differentiated empirical evidence for understanding the psychological significance of fertility motivations at different stages of the childbearing process.

The present study further found that different types of fertility motivation exhibited distinct patterns of association with short-term fertility plans. In the overall sample, Traditional Utility Motivation was significantly and positively associated with plans to have a child within the next 2 years, whereas the overall associations of Emotional Fulfillment Motivation and Family Responsibility Motivation were not statistically significant. This pattern is broadly consistent with previous research in China showing that different fertility motivations have distinct implications for fertility (Zheng et al., 2005; Chen and Hu, 2020; Song and Hu, 2022). With socioeconomic development, the emotional value of children, including parent–child companionship, emotional bonds, and family warmth, has received increasing emphasis (Kagitcibasi and Ataca, 2005; Yang and Wu, 2021). However, attaching importance to these values does not necessarily correspond to near-term fertility plans. By contrast, Traditional Utility Motivation encompasses relatively explicit instrumental goals, such as family continuity, old-age support, and the economic functions of children. From a goal-oriented perspective, such value orientations may be more readily linked to concrete fertility plans (Locke and Latham, 2002; Gollwitzer, 1999). However, the associations between fertility motivations and short-term fertility plans were not consistent across different stages of family formation. Further analyses by parity revealed heterogeneity in these associations across different types of fertility motivations.

For Emotional Fulfillment Motivation, **the main analysis showed that** its association with short-term fertility plans was primarily observed among childless adults: Emotional Fulfillment Motivation was significantly and positively associated with short-term fertility plans among childless adults, whereas no statistically significant association was found among adults with one child or those with two or more children. The transition to first-time parenthood involves not only the addition of a new family member but also the acquisition of a parental identity and the emergence of the parent–child relationship as a new life experience (Nelson et al., 2014). Thus, for individuals who have not yet entered parenthood, expectations of parent–child companionship, watching a child grow, and the experience of becoming a parent may have more direct relevance to fertility decision-making. Previous research in China has found that fertility values centered on personal experience and emotional fulfillment do not necessarily correspond to higher overall fertility levels (Chen and Hu, 2020). The present study further suggests that this association may differ according to whether individuals have already entered parenthood: Emotional Fulfillment Motivation was primarily associated with first-birth plans among childless adults, whereas no significant association was observed among parents. One possible explanation is that existing parent–child relationships may already provide parents with some of the emotional experiences associated with childbearing; consequently, the same emotional needs may not necessarily correspond to plans for another birth in the near term. This interpretation is consistent with previous research suggesting that childbearing experience may alter the relationship between the value of children and subsequent fertility (Nauck, 2014). It should be noted that these parity differences remained supported in robustness analyses using household-wave clustering and retaining only one respondent per household-wave. However, when the analysis was restricted to respondents with valid survey weights, the joint test did not reach statistical significance, regardless of whether survey weights were applied. The parity heterogeneity in Emotional Fulfillment Motivation should therefore be interpreted with caution.

Traditional Utility Motivation exhibited more pronounced differences across parity groups. It was negatively associated with short-term fertility plans among childless adults but positively associated with such plans among parents, suggesting that the fertility implications of traditional utility values may differ according to prior childbearing experience. Traditional Utility Motivation primarily encompasses values related to lineage continuation, old-age support, and the economic functions of children. In the context of intensifying educational competition, rising investments in childrearing, and substantial uncertainty surrounding children’s future development (Xie et al., 2023), stronger instrumental expectations among childless adults may also entail greater consideration of childrearing costs and uncertainty as to whether these expected functions of children can ultimately be realized, and therefore may not correspond to more positive first-birth plans. Previous research has similarly found that although traditional beliefs such as lineage continuation are positively associated with preferences for a larger number of children, this relationship is constrained by expectations regarding children’s future achievements (Zhang and Zhang, 2024). By contrast, among individuals who are already parents, values such as family continuity and intergenerational support are grounded in actual childbearing and childrearing experiences, and Traditional Utility Motivation was positively associated with plans for further childbearing. This finding is consistent with previous research showing that the association between the value of children and subsequent fertility varies according to the number of children individuals already have (Nauck, 2014). Thus, the present study further indicates that Traditional Utility Motivation does not have uniform fertility implications across groups; rather, its association with short-term fertility plans differs according to individuals’ parity.

## 6 Limitations and future research

This study has several limitations. First, fertility motivations and short-term fertility plans were measured within the same survey wave. Therefore, their temporal ordering cannot be established, and no causal inferences can be drawn from the observed associations. The TDIB model was used only as a conceptual framework for understanding the relationship between fertility motivations and short-term fertility plans; the full psychological sequence described by the model was not examined longitudinally. Future research could use longitudinal data to further investigate the prospective relationships between fertility motivations and subsequent fertility decision-making and actual fertility behavior. Second, when the analysis was restricted to respondents with valid survey weights, the joint test of the Emotional Fulfillment Motivation × parity interaction did not reach the 5% significance level, and the result remained essentially unchanged when survey weights were subsequently applied to the same sample. Therefore, the evidence for parity heterogeneity in Emotional Fulfillment Motivation should be interpreted with caution and warrants further examination in other representative samples. Third, the fertility motivation module followed different questionnaire routing rules in the 2020 and 2022 survey waves. The 2022 wave primarily supplemented respondents who had not completed the module in 2020, and the sample composition therefore differed between the two waves. The wave-stratified and cross-wave robustness analyses found no significant differences across survey waves for Emotional Fulfillment Motivation or Traditional Utility Motivation, whereas Family Responsibility Motivation exhibited some cross-wave heterogeneity. These findings should therefore be further validated using data collected under a consistent survey design. Fourth, owing to the design of the CFPS questionnaire, the study population was primarily restricted to married or cohabiting adults aged 22–49, with eligibility for the fertility motivation module further determined by age and questionnaire routing criteria. The findings therefore apply primarily to the partnered population defined in this study and should not be generalized directly to the entire Chinese population of reproductive age.

## Data Availability

Publicly available datasets were analyzed in this study. The data are from the China Family Panel Studies (CFPS) and are available to researchers upon application to the Institute of Social Science Survey (ISSS), Peking University: https://www.isss.pku.edu.cn/cfps/en/.
