# Supplementary material for: Psychological fertility motivations and short-term fertility plans among partnered Chinese adults: differences across parity groups
Source: Front Psychol. 2026 Oct 1;17:1917528. doi: 10.3389/fpsyg.2026.1917528 (PMC13669190; doi:10.3389/fpsyg.2026.1917528)
Supplement: Supplementary file 1 [file Table_1.DOCX]

**Supplementary Table S1. Sample Selection and Construction of the Final Analytical Sample**

| **Sample-selection step** | **2020 excluded, N** | **2020 remaining, N** | **2022 excluded, N** | **2022 remaining, N** |
| --- | --- | --- | --- | --- |
| Initial adult sample | — | 28,530 | — | 27,001 |
| Excluded: all nine fertility motivation items structurally unobserved (coded as “not applicable”) | 4,175 | 24,355 | 18,105 | 8,896 |
| Excluded: refusal, “don’t know,” or other invalid values on the fertility motivation items | 159 | 24,196 | 31 | 8,865 |
| Excluded: fertility plan structurally unobserved (coded as “not applicable”) | 11,792 | 12,404 | 3,300 | 5,565 |
| Excluded: refusal, “don’t know,” or other invalid values on the fertility plan item | 93 | 12,311 | 29 | 5,536 |
| Excluded: shared system-missing cases across all nine fertility motivation items and the fertility plan variable (coded as “.”), total | 3,448 | 8,863 | 3,336 | 2,200 |
| Of which: structural exclusion outside the study age range (aged ≤21 or ≥50) | 2,375 | — | 2,492 | — |
| Of which: shared system-missing cases of undetermined cause within the study age range (aged 22–49) | 1,073 | — | 844 | — |
| Excluded: missing or invalid parity information | 0 | 8,863 | 1 | 2,199 |
| Excluded: missing data on covariates | 218 | 8,645 | 168 | 2,031 |
| Wave-specific complete-case analytical sample | — | 8,645 | — | 2,031 |
| Excluded after pooling: respondents appearing in both waves (2020 record retained) | — | — | 5 | 10,671 |
| **Final analytical sample** | **—** | **8,645** | **—** | **2,026** |

**Note.** Shared system missingness refers to cases in which all nine fertility motivation items and the fertility plan variable were simultaneously system-missing. Five respondents appeared in both waves; their 2020 records were retained, and the corresponding 2022 records were excluded.
